# Risk factor analysis and outcomes of heart transplant recipients infected by COVID-19

**DOI:** 10.3389/fimmu.2025.1597333

**Published:** 2025-07-30

**Authors:** Lin Zhang, Sipeng Chen, Shanshan Zheng, Sheng Liu, Chenfei Rao, Zhongkai Liao, Xiaonan Fang, Xiaoying Hu, Jie Huang, Zhe Zheng

**Affiliations:** ^1^ Department of Cardiovascular Surgery, Fuwai Hospital, National Center for Cardiovascular Diseases, Chinese Academy of Medical Sciences and Peking Union Medical College, Beijing, China; ^2^ National Clinical Research Center for Cardiovascular Diseases, Fuwai Hospital, Chinese Academy of Medical Sciences, Beijing, China

**Keywords:** heart transplantation, COVID-19 infection, omicron variant, severity rate, risk factors, COVID-19 vaccine, sirolimus

## Abstract

**Objective:**

This study aims to evaluate the prevalence, clinical characteristics, severity, mortality, and outcomes of COVID-19 infection in heart transplant recipients, focusing on risk factors for severe disease.

**Methods:**

A retrospective, observational study was conducted on adult heart transplant patients (HTxs) at Fuwai Hospital from December 1, 2022, to February 28, 2023, with follow-up until May 30, 2024. Clinical data were collected via telephone surveys and medical records. Logistic regression analyses were conducted to explore risk factors for severe disease.

**Results:**

In total, 728 of the 916 HTxs were infected with COVID-19 (79.48%); the vaccination rate was 27.95%. Of infected cases, 56.18% were mild, 18.82% moderate, 19.26% severe, and 5.77% critical. Severe disease occurred in 25.00%, with a mortality rate of 4.54%. Logistic regression analyses revealed that age (OR 1.048, 95% CI 1.031-1.066, P<0.001), history of diabetes (OR 1.829, 95% CI 1.221-2.740, P=0.005), Chronic kidney disease stage≥3 (OR 2.557, 95% CI 1.650-3.963, P<0.001) and immunosuppressive regimens including sirolimus (OR 1.639, 95% CI 1.145-2.348, P=0.007) were independent risk factors for severe infection, while age (OR 1.102, 95% CI 1.053-1.154, P<0.001) and Chronic kidney disease stage≥3 (OR 6.342, 95% CI 2.980-13.499, P<0.001) were independent risk factors for post-infection mortality. COVID-19 vaccination (OR 0.169, 95% CI 0.039-0.733, P=0.018) was found to be a protective factor against post-infection mortality.

**Conclusion:**

COVID-19 vaccination is recommended for HTxs to reduce severe outcomes and mortality. Sirolimus use was independently associated with severe infection, highlighting the need for careful management of immunosuppression.

## 1 Introduction

Since the onset of the global pandemic in early 2020, there have been over 662 million confirmed cases of COVID-19 as reported by the World Health Organization by March 2024 (World Health Organization. Coronavirus (COVID-19) dashboard. https://covid19.who.int). Heart transplant recipients(HTxs), due to their chronic use of immunosuppressive medications and prevalent comorbidities, are thought to be at a significantly higher risk of contracting SARS-CoV-2 and experiencing severe outcomes (1). Multiple studies have demonstrated that SARS-CoV-2 infection in this patient population is associated with high rates of case fatality and hospitalization (2–5).

The emergence of the Omicron variant in late 2021 marked a significant turning point in the pandemic’s course. Although the Omicron variant has shown higher transmissibility, research indicates that its symptoms and outcomes are generally less severe than those associated with earlier variants (6, 7).

However, data specific to HTxs and the long-term effects of Omicron in this population remain sparse and unclear. This study seeks to fill this gap by conducting the largest single-center analysis to date, examining the clinical characteristics, risk factors, disease progression, and follow-up outcomes of heart transplant recipients infected with Omicron. The study also reviews the protective effects of COVID-19 vaccination in this population, specifically focusing on mortality reduction. Additionally, the impact of different immunosuppressive therapies on COVID-19 outcomes in HTxs is a central point of analysis.

## 2 Methods

### 2.1 Research design and methodology

This single-center, retrospective, observational study conducted in a real-world setting at Fuwai Hospital, Beijing, China. It focused on HTxs affected by COVID-19 during the prevalence of the Omicron variant. The enrollment period was from December 1, 2022, to February 28, 2023, coinciding with the peak of the Omicron variant wave.

Inclusion Criteria: The study included adult patients (≥18 years old) who had undergone heart transplantation at least one month before enrollment. The informed consent of deceased patients is the consent of their family members. The remaining patients provided informed consent on their own. Before collecting information from patients who are followed up by phone, an informed consent was read out, and only after obtaining consent can information be collected. For deceased recipients, informed consent was obtained from their family members. Exclusion Criteria: The study excluded patients who declined participation, those who had passed away before the start of the study, and those infected with SARS-CoV-2 prior to heart transplantation.

The study followed the ethical guidelines outlined in the Declaration of Helsinki (1975 revision) and Strengthening the Reporting of Observational Studies in Epidemiology (STROBE) guidelines. The study was approved by the Fuwai Hospital Ethics Committee (Approval No. 2023-1955). All transplanted organs were obtained voluntarily and without coercion, with documented informed consent from donors. The study complies with the Declaration of Istanbul and with relevant national and institutional ethical standards. For all telephone interviews, verbal informed consent was obtained following an approved consent process, and all consent procedures were recorded and archived in accordance with the requirements of the institutional ethics committee. Data handling complied with institutional and national privacy regulations. Regular follow-up was conducted for recipients diagnosed with COVID-19 infection, until March 31, 2024.

### 2.2 COVID-19 diagnosis

All patients were enrolled during the outbreak of the Omicron variant. Hospitalized patients and some home patients were confirmed to be infected with the Omicron variant through PCR. Some home patients were diagnosed using antigen testing and could not be determined to be infected with the Omicron variant. The classification of COVID-19 severity was based on the criteria outlined in the 10th Edition of China’s Diagnosis and Treatment Protocol for COVID-19 (8). The mild disease cases were those only got upper respiratory tract infection symptoms, such as dry throat, sore throat, cough, or fever. Moderate disease cases were those persistent high fever for more than 3 days or (and) coughing, shortness of breath, but respiratory rate (RR)<30 breaths/min, and oxygen saturation greater than 93% when inhaling air in a resting state. Imaging findings showed characteristic pneumonia caused by COVID-19. Severe cases characterized by significant respiratory distress (respiratory rate ≥30 breaths/min, oxygen saturation <93%, or worsening lung lesions, PaO2/FiO2 ≤300 mmHg). Critical cases were defined as those involving respiratory failure necessitating mechanical ventilation, shock, or multi-organ failure, requiring intensive care unit (ICU) admission.

### 2.3 Data collection, vaccination, and follow-up

Data collection was conducted via telephonic surveys and comprehensive medical record analysis. For all included cases, hospitalization, ICU admission, mortality, and biopsy-proven rejection were validated through institutional medical records whenever patients received care in either outpatient or inpatient settings. Only for patients who did not seek medical care at any healthcare facility were telephone interviews used to obtain clinical information. Data collected from phone interviews was confirmed with inpatient medical record data. The confirmed data includes: vaccination status, the type of vaccines used, dosage, inoculation times, and outcomes. A specialized online data collection platform was utilized to gather demographic data including age, gender, weight, and body mass index; medical history related to heart transplantation; and medication information including immunosuppressive regimens and other combined medications. All enrolled patients in our study received inactivated COVID-19 vaccines; no mRNA vaccines were administered. Vaccination status was classified as unvaccinated, 1 dose, 2 doses, or ≥3 doses. This reflects national vaccine availability during the study period in China, where inactivated vaccines were predominantly used. The primary endpoints were pre-specified and included: the incidence rate, mortality rate, and severity rates (defined as a composite endpoint comprising all-cause mortality and cases meeting the criteria for severe or critical disease, including ICU admission) of novel coronavirus infection post-heart transplantation. Secondary outcomes included vaccination status, hospitalization rates, and biopsy-proven rejection during follow-up. The follow-up period continued until death or the end of the study.

Data extraction focused on variables associated with COVID-19 severity and mortality, such as age, gender, BMI, transplantation etiology, smoking history, hypertension, diabetes, cerebrovascular and chronic respiratory diseases, chronic kidney disease (CKD), vaccination history, and immunosuppressive regimen adjustments during the infection phase. Univariate and multivariate analyses were applied based on these variables. Due to the small number of severe outcomes in individual dose groups, vaccination was analyzed as a binary variable (vaccinated *vs*. unvaccinated) in multivariable logistic regression models. Time-to-event analyses, such as Kaplan-Meier curves or Cox proportional hazards models, were not feasible in this study due to the short interval between infection and clinical outcomes and the uncertainty in defining the exact onset time. Logistic regression was therefore used to model binary outcomes.

### 2.4 Statistical methodology

Continuous variables were summarized as means (± SD) or medians (IQR), and categorical variables as frequencies (percentage). Univariate comparisons were conducted using the χ^2^ test or Fisher exact test for categorical variables, and the Student’s t-test or Mann-Whitney U-test for continuous variables, as appropriate. Logistic regression models were used to calculate odds ratios (ORs) with corresponding 95% confidence intervals (CIs). Variables with a P-value < 0.05 in univariate analysis or those deemed clinically relevant were included in multivariable logistic regression models to identify independent predictors. A two-sided P < 0.05 was considered statistically significant. Analyses were performed with SAS version 9.4 (SAS Institute, Cary, NC, USA).

## 3 Results

### 3.1 Baseline characteristics

This study included 916 adult heart transplant recipients. The mean age was 52.01± 13.20 years, and 215 (23.47%) were female. During the enrollment period, which spanned from December 1, 2022, to February 28, 2023, 728 (79.48%) patients developed COVID-19, as detailed in **Table 1**. 27.95% (256/916) of the HTxs were vaccinated. Among the COVID-19 group, hypertension(431, 59.20%)was the most prevalent comorbidity, followed by diabetes mellitus (423, 58.10%), chronic kidney disease stage≥3 (97, 16.00%).

**Table 1:** Demographic and clinical characteristics of the study participants.

Variable	Total (n = 916)	Infection group (n = 728)	Non infection group (n = 188)	P value
Age, mean (SD), y	52.01 ± 13.20	51.80 ± 12.97	52.87 ± 14.03	0.318
Female, n (%)	215 (23.47)	164 (22.56)	51 (26.98)	0.184
BMI, mean (SD), kg/m^2^	24.44 ± 3.76	24.49 ± 3.76	24.23 ± 3.79	0.388
Time since HT, mean (SD), y	6.64 ± 4.53	6.67 ± 4.46	6.49 ± 4.80	0.633
Comorbidities, n (%)				
Smoking	512 (55.90)	413 (56.73)	99 (52.66)	0.316
Diabetes	544 (59.39)	423 (58.10)	121 (64.36)	0.119
Hypertension	540 (58.95)	431 (59.20)	109 (57.98)	0.761
CKD stage≥3	143 (15.66)	97 (16.00)	27 (14.36)	0.582
Cancer	13 (1.42)	11 (1.51)	2 (1.06)	0.638
Cerebrovascular disease	59 (6.44)	49 (6.74)	10 (5.29)	0.470
Respiratory	63 (6.88)	55 (7.57)	8 (4.23)	0.107
Etiology, n (%)				0.900
Cardiomyopathy	729 (79.59)	583 (80.19)	146 (77.25)	
Coronary artery disease	127 (13.86)	98 (13.48)	29 (15.34)	
Valvular disease	27 (2.95)	20 (2.75)	7 (3.70)	
Congenital heart disease	20 (2.18)	15 (2.06)	5 (2.65)	
Others	13 (1.8)	11 (1.9)	2 (1.4)	
Immunosuppressants, n (%)				
Tac	463 (51.33)	363 (50.56)	100 (54.35)	0.359
CsA	336 (38.58)	266 (38.72)	70 (38.04)	0.867
MMF	876 (97.12)	698 (97.21)	178 (96.74)	0.731
Steroids	887 (98.34)	707 (98.47)	180 (97.83)	0.544
Sirolimus	376 (41.69)	305 (42.48)	71 (38.59)	0.339
COVID-19 vaccine, n (%)	256 (27.95)	203 (27.88)	53 (28.19)	0.974
Dose				0.447
Unvaccinated	660 (72.05)	525 (72.12)	135 (71.81)	
1 doses	40 (4.37)	33 (4.53)	7 (3.72)	
2 doses	92 (10.04)	77 (10.58)	15 (11.72)	
≥3 doses	124 (13.54)	93 (12.77)	31 (24.22)	

Data are mean ± SD or median (interquartile range [IQR]) or n (%).

BMI, body mass index; HT, heart transplant; CKD, Chronic kidney disease; Tac, tacrolimus; CsA, cyclosporin A; MMF, mycophenolate sodium enteric-coated tablets or mycophenolate mofetil.

### 3.2 Clinical outcomes of COVID-19 infection

Among the 728 infected HTxs, the severity was categorized as mild (409, 56.18%), moderate (137, 18.82%), severe (140, 19.26%), and critical (42, 5.77%), with the rate of severe illness (severe and critical combined) being 25.00% (182/728), as detailed in **Table 2**. The hospitalization rate was 27.06% (197/728), with 17.61% (128/728) requiring ICU treatment. The mortality rate was 4.54% (34/728).

**Table 2:** COVID-19 infections and outcomes among heart transplant recipients.

Characteristics	Total (n = 728)
Time since HT, M (Q25,Q75), y	5.81 (2.90, 9.11)
Time since HT<1,	26 (10.45)
Time since HT≥1,	651 (89.55)
Symptoms, n (%)	
Asymptomatic	10 (1.38)
Fever	612 (84.18)
Sore throat/dry throat	288 (39.61)
Fatigue	467 (64.24)
Myalgia	409 (56.26)
Cough	466 (64.10)
Nasal congestion/rhinorrhea	161 (22.15)
Gastrointestinal discomfort	182 (25.03)
Loss of taste/smell	199 (27.37)
Palpitations	88 (12.10)
Chest tightness, dyspnea	162 (22.28)
Duration of Symptoms, n (%)	
Asymptomatic	7 (0.96)
1–7 days	200 (27.51)
8–14 days	129 (17.74)
More than 14 days	391 (53.78)
Highest Level of Medical Support During COVID-19 Infection, n (%)	
Home observation	339 (46.63)
Outpatient	192 (26.41)
Emergency	27 (3.71)
Inpatient	158 (21.74)
ICU	128 (17.61)
COVID-19 Infection Classification, n (%)	
Mild	409 (56.18)
Moderate	137 (18.82)
Severe	140 (19.26)
Critical	42 (5.77)
Immunosuppressive Adjustments During Infection, n (%)	
CNI (tacrolimus, cyclosporine) dose reduction or discontinuation	312 (42.92)
MMF dose reduction or discontinuation	446 (61.35)
Sirolimus dose reduction or discontinuation	151 (20.77)
Dexamethasone use	102 (14.01)
Namatevir/Ritonavir	33 (4.53)
Azvudine	5 (0.69)
Symptom Outcomes Two Months Post-COVID-19 Infection	
Recovered with no residual symptoms	567 (77.99)
Persistent symptoms related to COVID-19 infection	160 (22.01)
Death	34 (4.54)

HT, heart transplant; ECMO, Extracorporeal membrane oxygenation; ICU, Intensive care unit.

The clinical characteristics of COVID-19 in HTxs did not significantly differ from those in non-transplanted patients (9), with the most common symptoms being fever (612/728, 84.18%), fatigue (467/728, 64.24%), cough (466/728, 64.10%), and myalgia (409/728, 56.26%). However, HTxs experienced prolonged durations of infection, with 53.78% (391) exceeding 14 days.

### 3.3 Risk factors for severe infection and fatal cases among infected patients

The study analyzed demographic and clinical data to identify risk factors for severe COVID-19 infection and mortality among HTxs. Key variables evaluated included age, sex, body mass index, vaccination status, comorbidities, and immunosuppressive regimens. Comparisons were made between recipients with severe infections and those with non-severe infections, as well as between survivors and non-survivors, as detailed in **Tables 3**, **4**.

**Table 3:** Analysis of risk factors for severe disease.

Variable	Non-severe (n = 546)	Severe (n = 182)	P value	Multivariate analysis		
				Adjusted OR	95% CI	P value
Age, mean (SD), y	49.91 ± 13.2	57.54 ± 10.25	<0.001	1.048	1.031-1.066	<0.001
Female, n (%)	123 (22.53)	41 (22.53)	1.000			
BMI, mean (SD), kg/m^2^	24.53 ± 3.62	24.38 ± 4.19	0.653			
Time since HT, mean (SD), y	6.58 ± 4.57	6.94 ± 4.08	0.355			
Comorbidities, n (%)						
Smoking	303 (55.49)	110 (60.44)	0.244			
Diabetes	285 (52.20)	136 (74.73)	<0.001	1.829	1.221-2.740	0.005
Hypertension	317 (58.06)	110 (60.44)	0.572			
CKD stage≥3	58 (10.62)	59 (32.42)	<0.001	2.557	1.650-3.963	<0.001
Cancer	9 (1.65)	1 (0.55)	0.465			
Cerebrovascular disease	37 (6.78)	11 (6.04)	0.730			
Respiratory	42 (7.69)	13 (7.14)	0.808			
Immunosuppressants, n (%)						
Tac	293 (54.26)	70 (39.33)	0.001			
CsA	212 (39.26)	54 (36.73)	0.578			
Sirolimus	211 (39.07)	94 (52.81)	0.001	1.639	1.145-2.348	0.007
COVID-19 vaccine, n (%)	164 (30.04)	39 (21.42)	0.024			

Data are mean ± SD or median (interquartile range [IQR]) or n (%).

BMI, body mass index; HT, heart transplant; CKD, Chronic kidney disease; Tac, tacrolimus; CsA, cyclosporin A; MMF, mycophenolate sodium enteric-coated tablets or mycophenolate mofetil.

**Table 4:** Analysis of risk factors for mortality.

Variable	Survival (n = 694)	Death (n = 34)	P value	Multivariate analysis		
				Adjusted OR	95% CI	P value
Age, mean (SD), y	51.24 ± 12.83	63.56 ± 10.14	<0.001	1.102	1.053-1.154	<0.001
Female, n (%)	158 (22.77)	6 (17.65)	0.674			
BMI, mean (SD), kg/m^2^	24.54 ± 3.76	22.01 ± 2.27	0.016			
Time since HT,mean (SD), y	6.57 ± 4.42	8.66 ± 4.75	0.008			
Comorbidities, n (%)						
Smoking	391 (56.34)	22 (64.71)	0.336			
Diabetes	399 (67.49)	24 (70.59)	0.131			
Hypertension	411 (59.22)	20 (54.82)	0.963			
CKD stage≥3	97 (13.98)	19 (61.38)	0.019	6.342	2.980-13.499	<0.001
Cancer	10 (1.44)	0 (0.00)	0.481			
Cerebrovascular disease	47 (6.77)	1 (2.94)	0.380			
Respiratory	55 (7.93)	0 (0.00)	0.088			
Immunosuppressants, n (%)						
Tac	354 (51.53)	9 (29.03)	0.014			
CsA	266 (38.72)	12 (38.71)	0.999			
Sirolimus	294 (42.79)	11 (35.48)	0.421			
COVID-19 vaccine, n (%)	201 (28.96)	2 (5.88)	0.003	0.169	0.039-0.733	0.018

Data are mean ± SD or median (interquartile range [IQR]) or n (%).

BMI, body mass index; HT, heart transplant; CKD, Chronic kidney disease; Tac, tacrolimus; CsA, cyclosporin A; MMF, mycophenolate sodium enteric-coated tablets or mycophenolate mofetil.

Age was an independent risk factor for both severe COVID-19 outcomes and mortality in HTxs. The odds ratio (OR) for severity was 1.048 (95% CI: 1.031-1.066, p < 0.001), and for mortality, it was 1.102 (95% CI: 1.053-1.154, p < 0.001). Diabetes was an independent risk factor for severe COVID-19 outcomes, with an OR of 1.829 (95% CI: 1.221-2.740, p = 0.005). CKD stage≥3, defined as an estimated glomerular filtration rate (eGFR) of less than 60 mL/min/1.73 m², was a significant risk factor for both severe COVID-19 outcomes (OR 2.577, 95% CI: 1.650-3.963, p < 0.001) and mortality (OR 6.342, 95% CI: 2.980-13.499, p < 0.001) in HTxs. Furthermore, the use of Sirolimus also emerged as an independent risk factor for severe COVID-19 outcomes, demonstrating an OR of 1.639 (95% CI: 1.145-2.348, p =0.007). Although tacrolimus use was associated with lower odds of severe outcomes and mortality, these associations did not reach statistical significance in multivariate analysis.

### 3.4 Impact of vaccination

The impact of vaccination on COVID-19 outcomes in HTxs was assessed. The overall vaccination rate was 27.95% (256/916). 4.37% (n = 40) of patients received one dose, 10.04% (n = 92) received two doses, and 13.54% (n = 124) received three or more doses (**Table 1**). Unvaccinated recipients showed significantly worse outcomes than vaccinated individuals, exhibiting higher mortality rates (6.10% *vs*. 0.99%, p = 0.005). Vaccination was associated with a substantial reduction in COVID-19 mortality risk, with an OR of 0.169 (95% CI: 0.039-0.733, p = 0.018, **Table 4**).

### 3.5 Long-term outcomes

The extended follow-up period for HTxs with COVID-19 infection spanned from March 1, 2023, to March 31, 2024. During this period, one death among study participants was attributed to acute rejection following COVID-19 infection, while four additional deaths resulted from other heart transplant-related complications, as detailed in **Table 5**. Acute rejection occurred in 16 (2.20%) of the infected HTxs, including 6 cases classified of Acute Cellular Rejection (ACR) grade 2R, 2 cases of ACR grade 3R, and 4 cases of Antibody-Mediated Rejection (AMR). Although the incidence of rejection was higher in the COVID-19 infection group than in the non-infected group (2.20% *vs*. 0.53%, P=0.087), this difference did not reach statistical significance.

**Table 5:** Outcomes one year after COVID-19 infection.

Characteristics	Total (n = 916)	Infection group (n = 728)	Non-infection group (n = 188)	P
Death	39	35	4	
Acute rejection	17 (1.86)	16 (2.20)	1 (0.53)	0.087
ACR	15 (1.64)	14 (1.92)	1 (0.53)	0.330
ACR 1R	6	6	0	
ACR 2R	7	6	1	
ACR 3R	2	2	0	
AMR	4 (0.44)	4 (0.55)	0 (0.00)	0.587
AMR 1	1	1	0	
AMR 2	3	3	0	

ACR, Acute Cellular Rejection; AMR, Antibody-Mediated Rejection.

## 4 Discussion

### 4.1 COVID-19 infections and outcomes

The heart transplant recipient’s cohort in our study was primarily infected with the SARS-CoV-2 Omicron BA.5.2 and BF.7 variants. Data concerning Omicron infections in HTxs remain limited and are based on small-scale studies. Our study represents the largest single-cohort analysis to date of HTxs, involving 916 participants from Fuwai Hospital. The infection rate was 79.48%, with severe infections occurring in 25.00% of cases and a mortality rate of 4.54%. Non-mRNA COVID-19 vaccines significantly reduced mortality among HTxs and had a protective effect against severe outcomes of COVID-19.

Initial data from the early stages of the pandemic indicated a high severe disease rate and mortality rate among HTxs who contracted COVID-19. A meta-analysis of 18 clinical studies conducted during the Delta variant period revealed that among a total of 5588 heart transplant patients, 2.54% contracted COVID-19. The likelihood of COVID-19 infection was markedly elevated in the HT population compared to the general population (OR 5.47; 95% CI 3.03–9.89, I2 = 90.6%, P < 0.001) (10). As the predominant strain shifted, the Omicron variant became the dominant strain, with higher transmissibility and infection rates compared to previous SARS-CoV-2 variants (11).

Although some studies suggest that the clinical presentation of HTxs may be atypical (4), our study found that the most common symptoms were fever (84.18%), fatigue (64.24%), cough (64.10%), and myalgia (56.26%), with asymptomatic patients accounting for only 1.38%. The clinical course did not significantly differ from that of non-transplanted patients (9).

Early studies during the Delta variant period showed a significantly higher risk among HTxs, with hospitalization rates ranging from 36.07% to 86.4%, and early mortality rates as high as 21%-28%, compared to 1%-5% in the general population (1–4, 10, 12–16). A meta-analysis of HTxs found a hospitalization rate of 82.9% (95% CI 77.1–87.9%; n = 242/298) and a pooled mortality rate of 27.6% (95% CI 23.2–32.2%; n = 107/384). Notably, the COVID-19 mortality rate in HTxs was significantly higher than in the general population (OR 3.37; 95% CI 2.25–5.05, I2 = 64.5%, P < 0.001) (10). However, several studies suggest that, after adjusting for comorbidities, the mortality risk for SOTr requiring hospitalization appears to be similar to that of the general population (1, 17, 18). As the pandemic evolved, some studies reported that the Omicron variants were associated with lower risks of severe outcomes in solid organ transplant recipients (SOTr) compared to the Delta and original strains (5–7, 19, 20). A recent study from Israel found that SOTr have significantly higher 30-day and 90-day mortality rates (2.4% *vs*. 0.8%, HR 3.42; P < 0.001 and 3.2% *vs*. 1.2%, HR 2.93; P < 0.001, respectively) and hospitalization rates (7.3% *vs*. 1.9%, HR 3.81; P < 0.001 and 7.6% *vs*. 1.9%, HR 4.11; P < 0.001) compared to matched controls during the Omicron variant predominant period (21). In our study, the hospitalization rate for HTxs was 27.06% (197/728), with 17.61% (128/728) requiring intensive care unit (ICU) treatment. The severe disease rate was 25.00% (182/728) with a mortality rate of 4.54% (34/728). The improvement in clinical outcomes may be related to the characteristics of the Omicron variant, increased access to treatment, and the protective effects of vaccination. In interpreting our findings, it is important to note that subgroup analyses for critical outcomes such as ICU admission and all-cause mortality were limited by the relatively small number of events. Consequently, observed trends should be interpreted with caution, and definitive conclusions regarding risk factors in these subgroups cannot be drawn from the current data.

### 4.2 Risk factors

Previous studies have identified several independent risk factors associated with severe COVID-19 outcomes, including advanced age, presence of multiple comorbidities, male gender, chronic kidney disease, and a history of cardiovascular diseases (22). Additionally, the use of immunosuppressants has been highlighted as a significant factor contributing to increased vulnerability among transplant recipients. Specifically, an analysis of 439 HTxs in the UNOS database demonstrated that increasing age, Black and Hispanic identity, diabetes, smoking history, and chronic steroid use at the time of heart transplantation were independently associated with COVID-19-related mortality (22). In our study, age emerged as a significant independent risk factor for both severe COVID-19 outcomes and mortality among HTxs. Comorbid diabetes was also independently associated with an increased risk of severe disease in this population. Notably, CKD was also independently associated with increased both severe COVID-19 outcomes and higher mortality in infected HTxs. These findings align with previous research, reinforcing the importance of close monitoring and tailored management strategies for HTxs with these risk factors to potentially improve clinical outcomes.

### 4.3 The effect of immunosuppressants

Our study found that the use of Sirolimus was independently associated with severe COVID-19 outcomes (OR = 1.639, 95% CI: 1.145-2.348, p = 0.007). Sirolimus is known to impair wound healing. In our cohort, it was prescribed only to clinically stable patients at least six months post-transplantation, primarily for indications such as renal dysfunction or prevention of cardiac allograft vasculopathy(CAV). Therefore, its association with COVID-19 outcomes is unlikely to be confounded by factors related to postoperative recovery.

Current research findings regarding mechanistic Target of Rapamycin (mTOR) inhibitors in the context of SARS-CoV-2 infection are contradictory (23–25). Early studies suggested that mTOR inhibitors like Sirolimus could reduce SARS-CoV-2 replication and suppress immune responses and cytokine storms, positing that it might be a potential strategy for mitigating the severity of COVID-19 (26–29). Some studies suggest that SARS-CoV-2 binds to Toll-like receptors (TLRs), activating the mTOR and NLRP3 (Nucleotide-binding oligomerization domain-like receptor family) inflammasome pathway, which leads to the production of IL-1β, a mediator of lung inflammation, fever, and fibrosis, and inducing pyroptosis (26). Sirolimus may prevent the progression of COVID-19 to severe forms by downregulating the senescence-associated secretory phenotype (SASP), mTOR-NLRP3-IL-1β axis, IL-6 pathway, and the number of senescent T cells (26).

Conversely, other research indicates that Sirolimus and its analogs may increase susceptibility to SARS-CoV-2 infection by activating lysosome-mediated immune suppression in human nasal epithelial cells and rodent models. Inhibiting the mTOR signaling pathway reduces intracellular immune responses, thereby enhancing vulnerability to the virus (25). Additionally, Sirolimus and certain rapalogs promote the translocation of TFEB (transcription factor EB) to the nucleus, inducing genes associated with lysosomal function and degrading antiviral factors like IFITM, thus facilitating viral entry into cells (25). A phase II, double-blind, placebo-controlled, multi-center randomized trial in high-risk adults hospitalized due to COVID-19 found that Sirolimus treatment did not reduce the risk of progression to advanced respiratory support or death (30). Furthermore, a study from France found that HTxs treated with Sirolimus (AOR = 2.71; 95% CI, 1.20–6.09) and everolimus (AOR = 1.24; 95% CI, 1.01–1.51) were at a higher risk of COVID-19 hospitalization compared to those receiving other immunosuppressive medications (31). These findings elucidate our results, indicating that Sirolimus is a risk factor for SARS-CoV-2 infection in the HTx population. While previous studies have indicated potential anti-inflammatory effects of Sirolimus through theoretical or mechanistic pathways, these were largely based on laboratory models rather than on clinical data. In contrast, our real-world study demonstrates an association between Sirolimus use and increased severity of COVID-19 in HTxs, underscoring the possible gap between theoretical benefits and clinical outcomes in this high-risk population. Thus, studies on Sirolimus highlight its dual role in SARS-CoV-2 infection, as it may alleviate inflammatory responses but simultaneously increase susceptibility to infection.

However, this association may be confounded by indication bias and patient selection, as mTOR inhibitors are frequently prescribed for recipients with underlying comorbidities such as chronic kidney disease or CAV—conditions that themselves have been associated with poorer COVID-19 outcomes (32, 33). This underscores the need for cautious, individualized use of Sirolimus in HTxs with COVID-19.

Our findings are broadly consistent with prior studies (30). While tacrolimus was not identified as an independent protective factor in multivariate analysis, we observed a trend toward lower severity and mortality among tacrolimus users, supporting previous reports of its potential immunomodulatory effects in mitigating COVID-19 severity—possibly through suppression of early T-cell activation and attenuation of the cytokine storm (34). We acknowledge the potential role of mycophenolic acid (MMF) in influencing COVID-19 outcomes. MMF may exacerbate disease severity by synergistically inducing lymphopenia with SARS-CoV-2 (35), thereby impairing antiviral immune responses. However, due to the near-universal use of MMF in our cohort (97.12%), we were unable to perform a meaningful statistical evaluation of its association with clinical outcomes. Future mechanistic studies and clinical trials are essential to better understand the impact of immunosuppressive therapies on COVID-19 outcomes in heart transplant recipients.

### 4.4 Vaccination

This study provides the first comprehensive evaluation of the impact of inactivated vaccines on HTxs. We observed that the overall vaccination rate among HTxs was 27.95%, lower than the 91.5% partial and 89.3% full vaccination coverage observed in the general population of China (36). Our results further support the correlation between SARS-CoV-2 inactivated vaccination and milder COVID-19 outcomes in HTxs. Unvaccinated HTxs faced a higher risk of mortality (6.10% *vs*. 0.99%, p = 0.005) compared to their vaccinated counterparts. Additionally, vaccination reduced the risk of COVID-19-related death (OR 0.169, 95% CI: 0.039-0.733, p = 0.018).

Despite the ability of Omicron variants to partially evade antibody-mediated neutralization, existing evidence demonstrates that inactivated vaccines remain effective in preventing severe disease, hospitalization, and death, even against Omicron (37, 38). Data from the China CDC, Hong Kong, Shanghai, and Singapore also indicated that inactivated vaccines provided protection against severe or critical illness (36, 37, 39–42).

Similarly, smaller studies involving HTxs demonstrated that a third dose of mRNA vaccines improved immune response rates to 53%, while a fourth dose led to 95% seroconversion in HTxs (43, 44). Although SOTr exhibit attenuated immune responses to COVID-19 vaccination compared to the general population, the protective effects increase with additional vaccine doses. A Canadian cohort study of 12,842 SOTr demonstrated a progressive increase in vaccine effectiveness with each dose: 31%, 46%, and 72% for the first, second, and third doses, respectively. Another Canadian study of 1,975 participants confirmed that three or more doses of mRNA vaccines significantly reduced disease severity (OR: 0.35, 95% CI: 0.21-0.60) (6). Similarly, smaller studies in HTxs showed that while two doses of mRNA vaccines resulted in low antibody responses (18%-48%), a third dose improved immune responses, with antibody rates increasing to 53%, and four doses led to 95% of HTxs developing immune responses (43, 45–49). In a retrospective study of 268 HTxs who had not been previously infected, receiving a third or fourth dose of mRNA vaccines significantly lowered the risk of COVID-19 infection (44). A separate study of 436 orthotopic HTxs found that vaccination reduced infection rates (19.7% *vs*. 48.6%, RR 0.41), hospitalizations (4.1% *vs*. 14.3%, RR 0.29), ICU admissions (1.1% *vs*. 4.3%), and deaths (0.8% *vs*. 4.3%, RR 0.19) compared to unvaccinated individuals (50). Based on these findings, we recommend that vaccines be considered part of the primary vaccination series for all HTxs without contraindications.

### 4.5 Transplantation rejection

Our study found that biopsy-proven acute rejection occurred in 16 (2.20%) of the infected HTxs, with a higher incidence in the COVID-19 infection group compared to the non-infected group (2.20% *vs*. 0.53%, P=0.09, **Table 3**), although this difference was not statistically significant. The severity of COVID-19 infection, the extent and duration of immunosuppression reduction, induction regimens, previous episodes of rejection, and the presence of donor-specific antibodies (DSA) may influence the risk of rejection in heart transplant (HT) recipients infected with COVID-19. However, data on the association between these factors and rejection risk in COVID-19-infected HTxs remain limited, with most reports being case studies (51–53). A review of 11 studies involving 1,179 kidney transplant recipients showed no significant association between COVID-19 and biopsy-proven rejection in controlled studies (P= 0.26) (54). However, non-controlled studies indicated a variable rejection incidence, with a pooled rate of 11.8% (54). In our study, due to the limited sample size and lack of detailed data on immunosuppression modification, we were unable to assess the causal relationship between COVID-19 and the risk of acute rejection. Overall, current evidence is insufficient to draw definitive conclusions regarding the impact of COVID-19 on rejection outcomes in transplant recipients. Further prospective, well-controlled studies are needed to clarify this question.

## 5 Limitations

This study has several limitations. As an observational study, it carries a potential risk of unmeasured confounding variables. Due to indication bias, patient selection, and treatment timing, the association between sirolimus use and severe COVID-19 outcomes may reflect underlying comorbidities that are themselves associated with poorer prognosis.

Moreover, while this study evaluated individual immunosuppressive agents, immunosuppressive regimens (e.g. dual *vs*. triple therapy) were not analyzed due to lack of structured data. Selection bias may be present, as the included participants may not fully represent the entire population of HTxs, thereby limiting the generalizability of the findings to other settings. Additionally, the specific SARS-CoV-2 variants and sublineages infecting our HTxs were not determined. All cases included in the final analysis were confirmed by PCR or antigen testing; no suspected COVID-19 cases were included. Furthermore, the study did not include HTxs in the perioperative period, as their characteristics and risks differ from those of stable patients, further limiting the generalizability of our results to this subgroup. The study may have been underpowered to detect associations in certain clinically relevant subgroups, particularly for rare but critical outcomes such as mortality. The small number of such events limited our ability to perform robust subgroup analyses. Future studies with larger multicenter cohorts are needed to validate these findings and improve generalizability. Moreover, as logistic regression was used to model binary outcomes, the study was unable to assess time-to-event relationships, and therefore could not evaluate temporal patterns or progression of clinical outcomes. Finally, as a retrospective study, there are inherent limitations in data accuracy due to reliance on medical records and telephone surveys, which may introduce recall bias. These limitations underscore the need for cautious interpretation of the findings and suggest that future multicenter studies with variant-specific analyses and a broader HTx population are warranted to enhance the robustness and applicability of these results.

## 6 Conclusion

Our study is the largest single-cohort analysis to date on COVID-19 infection results in heart transplant recipients, involving 916 participants. We observed an infection rate of 79.48%, with severe infections occurring in 24.86% of cases and a mortality rate of 4.54%. Inactivated COVID-19 vaccines significantly reduced mortality among HTxs and demonstrated a protective effect against severe COVID-19 outcomes. Furthermore, the use of sirolimus was independently associated with more severe COVID-19 outcomes. These findings underscore the importance of targeted vaccination strategies and the need for ongoing monitoring of high-risk populations, such as HTxs.

## Data Availability

The dataset generated and analyzed during the current study contains sensitive patient information and is not publicly available due to ethical and privacy restrictions. Access to the data may be granted upon reasonable request and with appropriate institutional review board (IRB) approval, in compliance with patient confidentiality regulations. Requests to access the datasets should be directed to ZZ, zhengzhe@fuwai.com.
